# Wrong place at the right time, a case of primary bronchial myoepithelial carcinoma

**DOI:** 10.1093/jscr/rjaf927

**Published:** 2025-11-25

**Authors:** Hermione Jemmett, Sanjeet Singh Avtaar Singh, Ella Bauwens, Katherine Quiohilag, Malcolm Will

**Affiliations:** Department of Cardiothoracic Surgery, Royal Infirmary of Edinburgh, 51 Little France Crescent, Old Dalkeith Road, Edinburgh, EH164SA, United Kingdom; Department of Cardiothoracic Surgery, Royal Infirmary of Edinburgh, 51 Little France Crescent, Old Dalkeith Road, Edinburgh, EH164SA, United Kingdom; University of Glasgow, School of Cardiovascular & Metabolic Health, University of Glasgow, University Avenue, Glasgow, G12 8QQ, United Kingdom; Department of Life Sciences and Medicine, King’s College London, Strand, London, WC2R 2LS, United Kingdom; Department of Pathology, Royal Infirmary of Edinburgh, 51 Little France Crescent, Old Dalkeith Road, Edinburgh, EH164SA, United Kingdom; Department of Cardiothoracic Surgery, Royal Infirmary of Edinburgh, 51 Little France Crescent, Old Dalkeith Road, Edinburgh, EH164SA, United Kingdom

**Keywords:** atypical lung cancer, minimally invasive surgery

## Abstract

Primary bronchial myoepithelial carcinoma is a rare neoplasm, accounting for less than 1% of all primary lung malignancies. These tumours typically arise from submucosal tracheobronchial glands and are resistant to chemotherapy and radiotherapy, making surgical resection the preferred treatment. We present the case of a 78-year-old male who was incidentally found to have a right upper lobe endobronchial lesion following investigation for a chest infection. Imaging revealed a 3.8 cm obstructive mass in the right main bronchus. Bronchoscopic biopsy confirmed a high-grade myoepithelial carcinoma. He underwent a serratus-sparing thoracotomy with a bronchial sleeve upper lobectomy and mediastinal lymphadenectomy. The anastomosis was reinforced with a pedicled thymo-pericardial diaphragmatic fat flap. Postoperative recovery was complicated by pneumonia, delirium, and electrolyte imbalance, all of which resolved with appropriate management. Histopathology confirmed complete resection. Follow-up imaging showed no recurrence. This case underscores the importance of early recognition and meticulous surgical management in treating this rare pulmonary malignancy.

## Introduction

Myoepithelial carcinomas are a rare subset of salivary neoplasms exclusively composed of myoepithelial cells. Cases of primary bronchial myoepithelial carcinoma are extremely rare, with fewer than 25 recorded cases [1]. These account for <1% of all primary lung malignancies [2]. They usually present as intrabronchial masses and probably originate from submucosal tracheobronchial glands [3]. This report presents a case of primary right main bronchial myoepithelial carcinoma in a 78-year-old male. Evidence suggests these tumours are not usually sensitive to radiation or chemotherapy. He had surgical resection via a thoracotomy and bronchial sleeve upper lobectomy, which was successful.

## Case report

**Figure 1:**
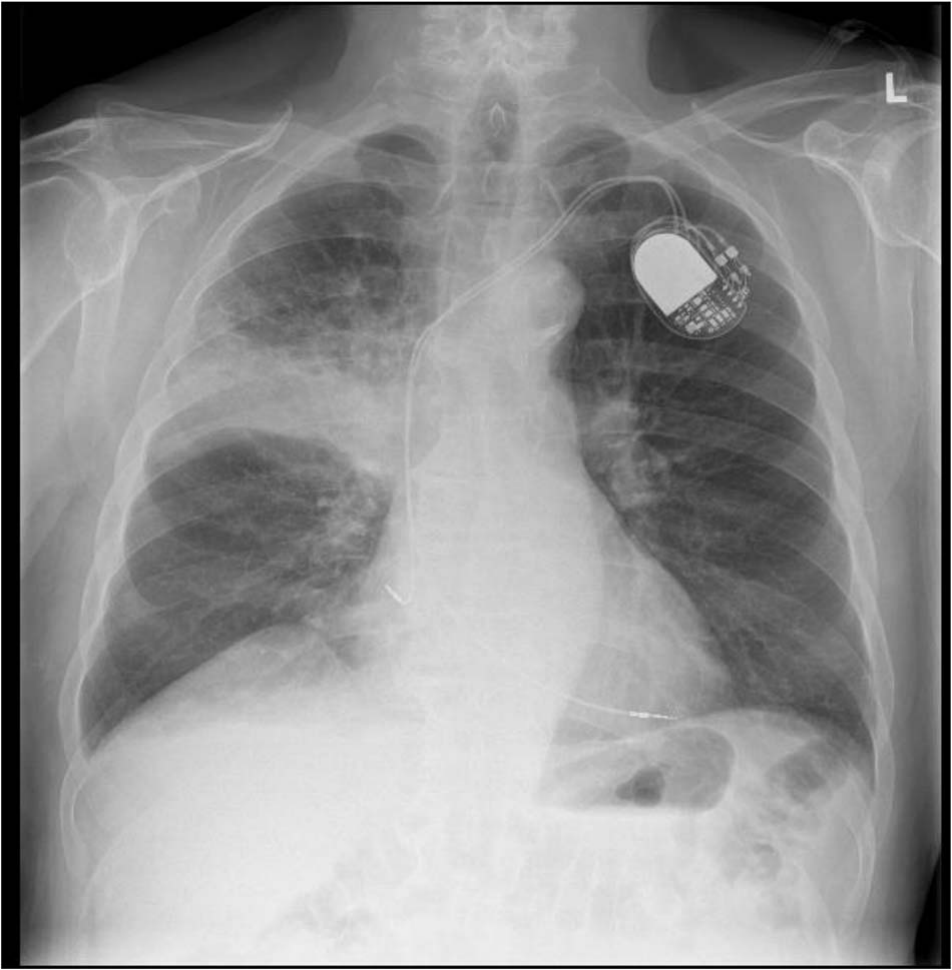
CXR showing right upper lobe consolidation with a small right pleural effusion (dual lead pacemaker noted).

**Figure 2:**
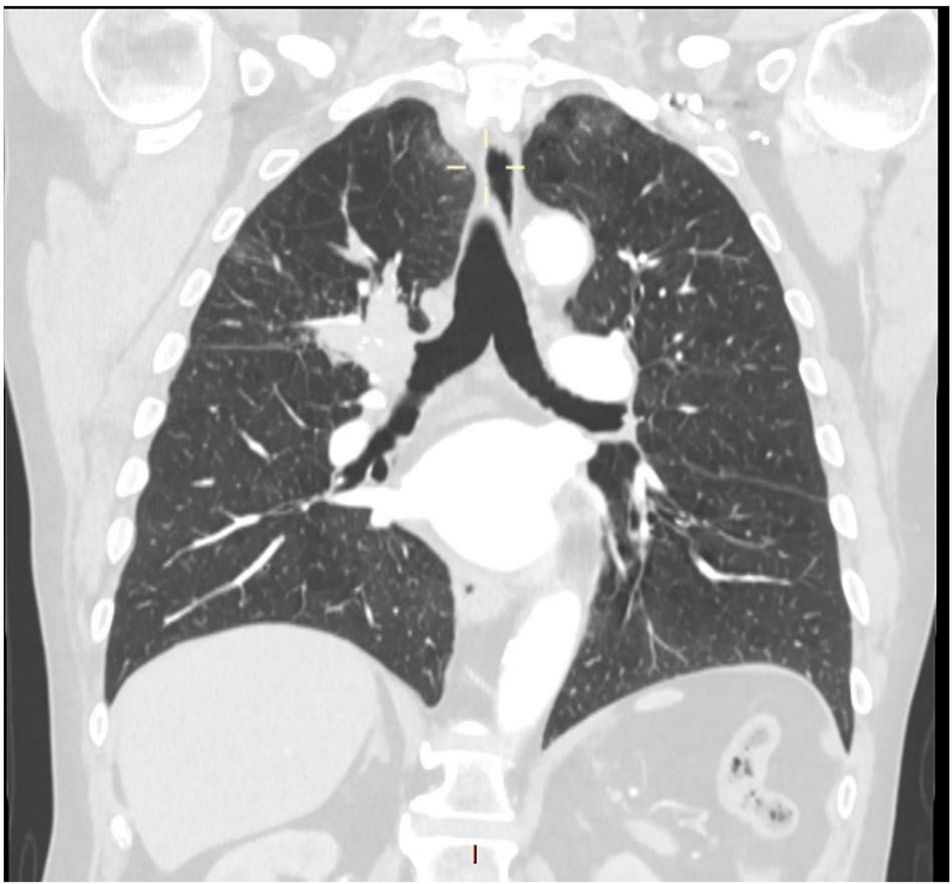
CT scan (coronal section) showing a large endobronchial lesion within the right upper lobe bronchus causing distal collapse of the right upper lobe.

**Figure 3:**
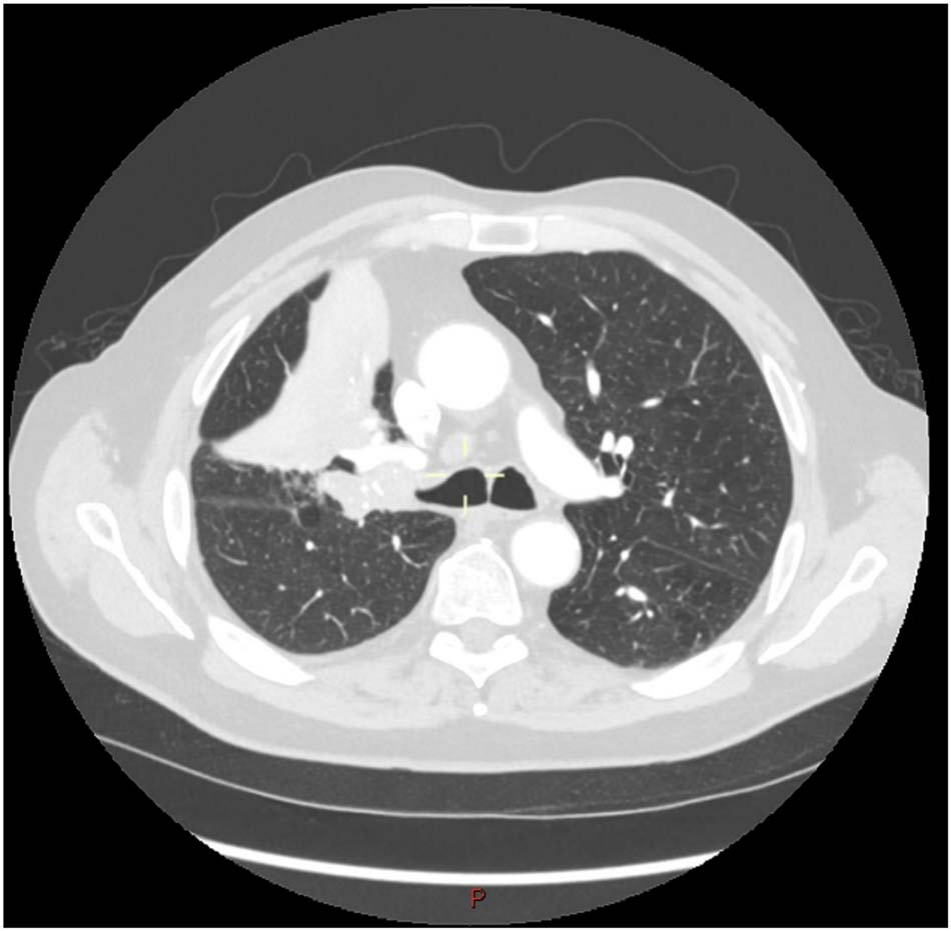
CT scan (transverse section) showing a large endobronchial lesion within the right upper lobe bronchus causing distal collapse of the right upper lobe.

**Figure 4:**
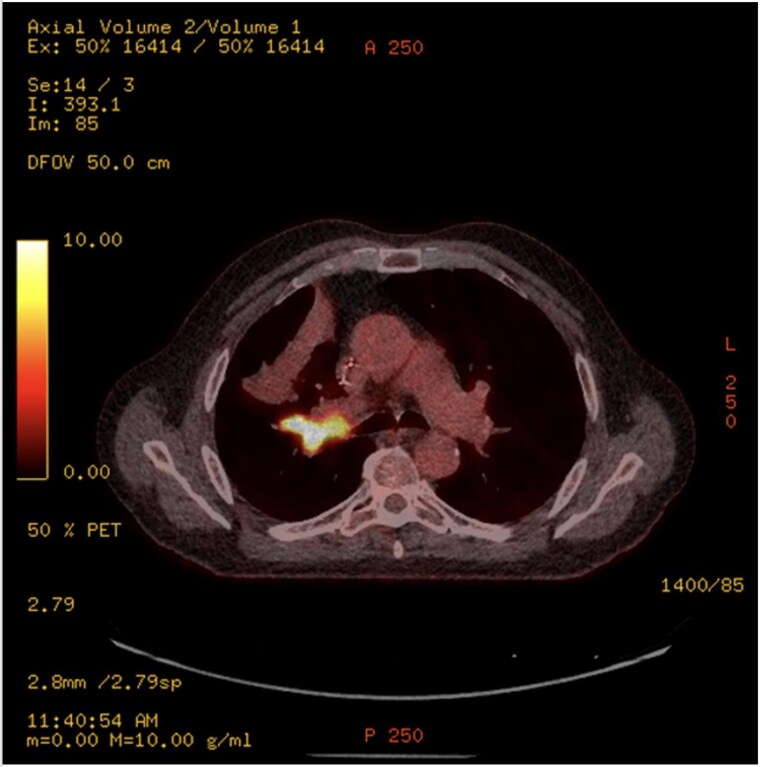
PET-CT showing an endobronchial lesion that measures 3.8 × 2 cm and is fluorodeoxyglucose avid (SUV max 13.8 g/ml).

This 78-year-old man initially presented with a chest infection in October 2024, where a chest X-Ray (CXR) showed right upper lobe (RUL) consolidation accompanied by a pleural effusion (Fig. 1). He had no other symptoms, including haemoptysis, shortness of breath, fever, anorexia, or weight loss. A follow-up computed tomography (CT) chest 6 weeks later revealed a large endobronchial lesion in the right main bronchus, resulting in consolidation and partial collapse of the RUL (Figs 2 and 3). He went on to have a positron emission tomography (PET) scan (Fig. 4), CT head, bronchoscopy, and endobronchial ultrasound (EBUS). Imaging revealed a 3.8 cm lesion in the right upper lobe bronchus, which was mostly occluded. The biopsies showed evidence of a myoepithelial carcinoma, a rare pulmonary neoplasm.

His relevant past medical history included asthma, transient ischaemic attack, atrial fibrillation with pulmonary vein ablation, AV node ablation, and permanent pacemaker insertion.

Pre-operatively, he had normal blood results. He underwent a flexible bronchoscopy, right video-assisted thoracoscopic surgery inspection, and thoracotomy with bronchial sleeve upper lobectomy in April 2025.

Bronchoscopy noted tumour occlusion of the right upper lobe bronchus orifice. After placement of intercostal blocks and a paravertebral catheter for analgesia, a serratus-sparing thoracotomy was performed in the fifth interspace. Nodes were dissected out, including the entire R2−4 nodal area, station 7, R10 inferior vein, R9, and R8. A hilar release manoeuvre was performed. The mass and 3 cm of the bronchus intermedius was removed. Frozen section on bronchial shavings showed no evidence of malignancy. An end-to-end anastomosis was performed with multiple interrupted 3-0 undyed polysorb sutures. Bronchoscopy confirmed patent middle and lower lobe orifices. A pedicled thymo-pericardial diaphragmatic fat flap was harvested and sutured to the lung staple line edge and the mediastinal pleura covering the anastomosis. The estimated blood loss was 150 ml.

In the High Dependency Unit, he developed a hospital-acquired pneumonia and subsequent delirium requiring a further course of intravenous antibiotics. His bloodwork post-operatively also highlighted a concurrent hyponatraemia and hyperkalaemia with a sodium of 127 and a potassium of 6.2. This was treated with furosemide and sodium zirconium. Osmolalities were normal, and all parameters returned to baseline. He was stepped down to the ward 5 days post-operatively. His CXR showed a right apical space as expected. His drain was removed on Day 7, and he was discharged home on post-operative Day 9. Pathology noted a firm, white, lobulated mass arising near the hilar resection margin with distal obstructive changes.

The tumour consists of cells arranged in a high-grade sheet, nested, and trabecular type patterns, with large areas of central necrosis in keeping with a high-grade myoepithelial carcinoma that was adequately resected.

**Figure 5:**
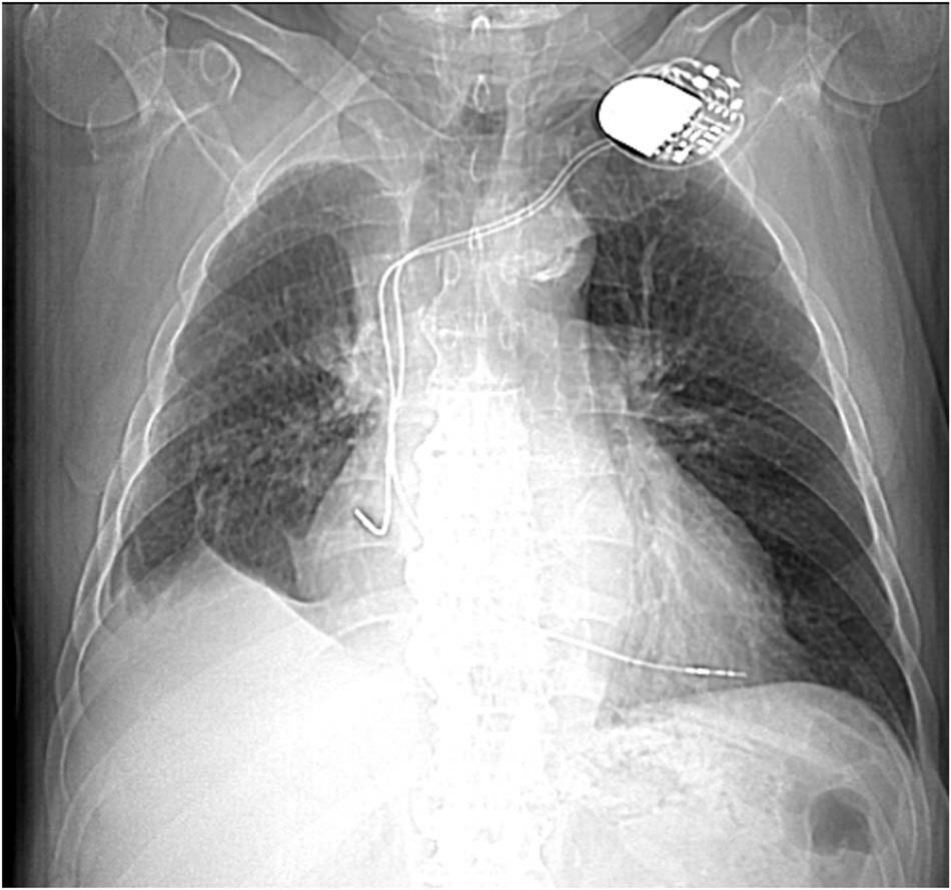
Scout CT scan showing postoperative volume loss in right hemithorax.

His post-operative clinic follow-up was satisfactory, with well-healed wounds and imaging showing volume loss but no sign of recurrence (Fig. 5).

## Discussion

Myoepithelial carcinoma was first described in the salivary glands in 1972. Owing to its origin from primitive myoepithelial cells, it may arise in both peripheral and central lung regions. There have been three other cases of sleeve resections for endobronchial myoepithelial carcinoma in the literature. (Table 1).

**Table 1:** Case reports in the literature describing endobronchial myoepithelial carcinoma

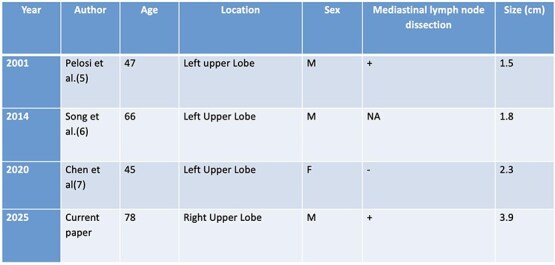

PET imaging may offer additional diagnostic insight. Rosen *et al*. [4] reported two cases with hypermetabolic activity [standardised update value (SUV) 6.6], consistent with our case which demonstrated an SUV of 13.8, suggesting high metabolic activity despite the tumour’s indolent histogenesis. However, the role of PET in staging and surveillance remains undefined due to limited data.

The imaging features of myoepithelial carcinoma of the lung are not well described in the literature. Routine imaging included chest X-ray, and CT scans were done in all the cases reviewed in the literature. On CXR, lesions typically appear as non-specific opacities or shadows. CT findings are more variable, ranging from smooth, well-circumscribed heterogeneous masses to solid spiculated or nodular lesions with irregular borders. This variability complicates preoperative differentiation from other bronchial neoplasms [4]. PET imaging may offer additional diagnostic insight. Rosen *et al*. [4] reported two cases with hypermetabolic activity (SUV 6.6), consistent with our case, which demonstrated an SUV of 13.8, suggesting high metabolic activity despite the tumour’s indolent histogenesis.

Microscopically, the tumour exhibited a high-grade architecture, with cells arranged in sheets, nests, and trabecular patterns. Notably, there were extensive areas of central necrosis, a feature previously reported in three other high-grade cases reviewed by Rosen *et al*. [4].

Immunohistochemical staining demonstrated strong positivity for AE1/3, with patchy expression of SOX10 and p40, and weak, occasional staining for S100. This staining profile aligns with findings by Chao *et al*. [1], who also noted variable S100 expression in pulmonary myoepithelial carcinomas. The immunophenotype supports the diagnosis and highlights the diagnostic challenge posed by this rare entity, particularly in small biopsy specimens.

Macroscopically, the majority of the tumours within the literature were described as well-circumscribed, nodular and had a solid white cut surface, similar to our case that had a firm white lobulated mass [4]. Microscopically, the tumour exhibited a high-grade architecture, with cells arranged in nests and trabecular patterns. Notably, there were extensive areas of central necrosis, a feature previously reported in three other high-grade tumours reviewed by Rosen *et al*. [4]. Immunohistochemical staining demonstrated strong positivity for AE1/3, with patchy expression of SOX10 and p40, and weak, occasional staining for S100. This staining profile aligns with findings by Chao *et al*. [1], who also noted variable S100 expression in pulmonary myoepithelial carcinomas.

Differentiation of myoepithelial tumours from other primary salivary-type neoplasms of the lung hinges on the absence of a ductal component, a feature that distinguishes them from mixed or pleomorphic adenomas [4]. To date, there is no firm criterion for the diagnosis of malignancy in myoepithelial tumours of the salivary gland, although factors such as capsular invasion, cellular atypia, and mitotic rate are often quoted. High-grade tumours, as noted in our patient, showed enlarged nuclei, nuclear pleomorphism, chromatin clumping, prominent nucleoli, and nuclear membrane irregularities [4].

These cytological hallmarks, combined with central necrosis and immunohistochemical staining patterns, support the diagnosis of a high-grade malignant phenotype and reinforce the importance of complete surgical excision.

## Conclusion

Myoepithelial carcinomas of the lung are a rare entity, with a variety of presentations described in the literature. Diagnosis is usually based on pathology, with imaging findings usually non-specific compared to other lung malignancies. The lack of diagnostic criteria is reflective of how rare the disease is, but surgical management is both safe and effective, with good outcomes noted.
